# Endothelial Function and Insulin Resistance in Early Postmenopausal Women with Cardiovascular Risk Factors: Importance of *ESR1* and *NOS3* Polymorphisms

**DOI:** 10.1371/journal.pone.0103444

**Published:** 2014-07-31

**Authors:** Ruth Clapauch, André Felipe Mourão, Anete S. Mecenas, Priscila A. Maranhão, Ana Rossini, Eliete Bouskela

**Affiliations:** 1 Laboratory for Clinical and Experimental Research on Vascular Biology (BioVasc), Biomedical Center, State University of Rio de Janeiro, Rio de Janeiro, Brazil; 2 Hospital da Lagoa, Endocrinology Sector, Health Ministry, Rio de Janeiro, Brazil; 3 Departamento de Bioquímica, IBRAG, State University of Rio de Janeiro, Rio de Janeiro, Brazil; II Università di Napoli, Italy

## Abstract

Cardiovascular benefits from estradiol activation of nitric oxide endothelial production may depend on vascular wall and on estrogen receptor alpha (*ESR1*) and nitric oxide synthase (*NOS3*) polymorphisms. We have evaluated the microcirculation *in vivo* through nailfold videocapillaroscopy, before and after acute nasal estradiol administration at baseline and after increased sheer stress (postocclusive reactive hyperemia response) in 100 postmenopausal women, being 70 controls (healthy) and 30 simultaneously hypertensive and diabetic (HD), correlating their responses to *PvuII* and *XbaI ESR1* polymorphisms and to *VNTR*, *T-786C* and *G894T NOS3* variants. In HD women, C variant allele of *ESR1 Pvull* was associated to higher vasodilatation after estradiol (1.72 *vs* 1.64 mm/s, p = 0.01 compared to TT homozygotes) while *G894T and T-786C NOS3* polymorphisms were connected to lower increment after shear stress (15% among wild type and 10% among variant alleles, p = 0.02 and 0.04). The G variant allele of *ESR1 XbaI* polymorphism was associated to higher HOMA-IR (3.54 *vs.* 1.64, p = 0.01) in HD and higher glucose levels in healthy women (91.8 vs. 87.1 mg/dl, p = 0.01), in which increased waist and HOMA-IR were also related to the G allele in *NOS3 G894T* (waist 93.5 *vs* 88.2 cm, p = 0.02; HOMA-IR 2.89 *vs* 1.48, p = 0.05). *ESR1 Pvull, NOS3 G894T* and *T-786C* polymorphism analysis may be considered in HD postmenopausal women for endothelial response prediction following estrogen therapy but were not discriminatory for endothelial response in healthy women. *ESR1 XbaI* and *G894T NOS3* polymorphisms may be useful in accessing insulin resistance and type 2 diabetes risks in all women, even before menopause and occurrence of metabolic disease.

## Introduction

Cardiovascular (CV) disease remains the leading cause of female mortality [1]. Women’s CV risk increases steadily after 60 years [2], i.e., ten years after menopause, potentially due to loss of estrogen cardio protective effects. However, studies examining hormone therapy (HT) in relation to CV events are contradictory, probably due to different vascular wall conditions, which can be affected by HT timing and also by co-morbidities [3]. Nowadays 28% of 45–64 years old American women present 2–3 chronic conditions, hypertension/diabetes (HD) being the second most prevalent dyad, representing 23.6% of the couplets [4]. Early postmenopausal simultaneously HD women would likely present a more advanced-for-age vascular impairment. Predicting HT CV effects in HD women would be particularly important, since their baseline CV risk may be higher than that of healthy early postmenopausal ones.

The central target of estrogen anti-atherosclerotic action is the endothelium, a monolayer of cells covering internally the vascular wall. It functions as a barrier, but, most importantly, produces nitric oxide (NO) by converting L-arginine to L-citrulline upon activation of endothelial nitric oxide synthase (eNOS) [5]. Other stimuli like sheer-stress after exercise physiologically activate eNOS genomic production, resulting in hyperemia due to NO vasodilatatory effect. NO modulates blood flow and pressure and has important anti-atherogenic effects on platelets and vascular smooth muscle cells. Endothelial dysfunction, i.e., the inability of the endothelium to liberate NO properly, is considered the first step of the atherosclerotic process and a predictor of CV disease; moreover, the lack of endothelial-derived NO is associated to vasospasm and vascular infarction [6], [7]. Estradiol (E2) has a major impact on the vasculature, enhancing the release of dilating factors such as NO, prostacyclin, and endothelium-derived hyperpolarizing factor (EDHF), and decreasing the vasoconstricting ones like endothelin and angiotensin II [8]. Both destruction of the endothelial layer and administration of NO inhibitors were shown to prevent estrogen vascular effects in rats [9]. Later on, a sequence of non-classical, non-genomic, E2 receptor membrane actions involving rapid signal-transduction pathways was detailed, including activation of phosphatidylinositol 3-kinase, protein kinase B (Akt) cascade generation and, by Gαi-protein interaction, long lasting estrogen-mediated eNOS phosphorylation and genomic NO production [10], [11], [12].

Estradiol actions are manifest through estrogen nuclear receptors alpha (ERα) and beta (ERβ), encoded by *ESR1* and *ESR2* genes, respectively [13], and one G-protein coupled receptor [8]. Most reports consolidated ERα as the main responsible for E2 endothelial effects [14]: eliminating ERα from endothelial cells resulted in abolishment of E2-induced NO release [15]; likewise, NO bioavailability and the consequent vasodilatation induced by E2 were abolished in ERα KO mice [11]. *ESR1* is located in chromosome 6p25.1 and encodes a 6.8-kilobase mRNA containing eight exons [16]. *PvuII* (rs2234693) and *XbaI* (rs9340799), mapped in intron 1, are the two most studied polymorphisms of *ESR1* associated to CV risk factors, such as dyslipidemia, insulin resistance, hypertension, central obesity and type 2 diabetes [17], [18], [19]. The *PvuII* polymorphism, caused by a C>T transition is located ≈0.4 kb upstream from exon 2 and the *XbaI* polymorphism, caused by a G>A transition, is located approximately 50 bp apart from the *PvuII* polymorphic site [20], [21]. Genetic influences on E2 vasodilatatory effect involve also the *NOS3* gene, located on chromosome 7q35–36 [22], from which few polymorphisms have proven functional importance. In this study, we focused in three *NOS3* variants: *VNTR* (27 bp TR), *T-786C* (rs2070744), and *G894T* (rs1799983). *VNTR* is characterized by variable tandem repeats of 27 bp in intron 4 (4a/b) and has been associated with variations in NO, nitrite, and nitrate plasma levels that may reflect eNOS activity [23]. *T-786C*, a T>C transition in the promoter region of the gene reduces its activity and affects eNOS protein expression and function [24], [25]. *G894T*, a G>T substitution at nucleotide 894 in exon 7, causes structural change of eNOS protein that down regulates eNOS activity [26], [27].

Microvascular endothelial function can be studied through nailfold videocapillaroscopy (NVC), a well-validated technique which measures vasodilatation (reactive hyperemia response) as an evidence of NO liberation after ischemia/reperfusion, provoked by an occlusion distal to the examined area, with subsequent release and consequent sheer-stress endothelial stimulation. In this study we have used this technique to assess endothelial function before and after acute estradiol administration in HD and healthy postmenopausal women and analyzed the responses in each group in relation to *ESR1* and *NOS3* polymorphisms, in order to identify genetic benefit/risk determinants of HT endothelial effects, both in healthy and CV risk patients.

## Patients and Methods

### Ethics Statement

This study was conducted according to principles expressed in the Declaration of Helsinki and was approved by Hospital da Lagoa Ethics Committee (Research protocol no. 02/2005). The participants were informed about the design of the study and possible risks and discomforts related to the experiment, and all of them signed a written informed consent, approved by the Ethics Committee.

### Patients

One hundred postmenopausal women were selected from the Gynecological Endocrinology Clinic (Hospital da Lagoa, Rio de Janeiro, RJ, Brazil). Patients were divided into 2 groups: HD (n = 30), defined as simultaneously hypertensive (blood pressure [BP] >140×90 mmHg in at least 3 occasions or known hypertension controlled with medication) coupled with altered fasting plasma glucose (>110 mg% in at least 2 occasions) or diabetes mellitus using oral hypoglycemic agents; and healthy (n = 70), normotensive (BP <140×90 mmHg) and normoglycemic (fasting plasma glucose <100 mg%). In addition, HD women should be <60 years old and have <10 years of hormone deprivation (defined as time since menopause *minus* years of HT). Points for age, previous total and HDL cholesterol, smoking status and systolic blood pressure through the Framingham Risk Score were used to select HD patients with a 10-year risk for coronary heart disease <1% [28]. In most postmenopausal women composing the control group, mean age and time since menopause were similar to HD, although a larger limit for age (above 60 up to 70 years in 13 patients) and years of hormone deprivation >10 years (in 3 women) were tolerated for inclusion, as long as these women remained healthy.

Exclusion criteria were BP >160/100 mmHg, previous CV event, thyroid dysfunction, use of Ca^++^-channel or β-blockers, sulfonylureas, corticosteroids, tranquilizers and soy isoflavones, evidence or suspicion of hormone dependent cancer and past venous thromboembolism. The procedure was fully explained to each patient. Women on current HT (n = 10 in HD group and n = 17 in C group) withhold it one month before NVC evaluation, scheduled at the Laboratory for Clinical and Experimental Research on Vascular Biology (BioVasc).

### Methods

On the day of the experiment, each patient arrived in the morning, after 12 hours fasting, had the blood sample collected and waited for 30 min, acclimatized at 24±1°C, during which she answered clinical questions and had her blood pressure and waist assessed by the same observer. From the same blood sample, hormone and inflammatory markers determinations were performed at Laboratório Diagnósticos da América; biochemical determinations at Hospital da Lagoa, according to methodology and reference values given below. Genetic determinations were performed in part of the total blood collected, maintained in EDTA tubes without heparin and stored in a −70°C freezer until DNA extraction.

Glucose and insulin were measured by enzymatic colorimetric method (reference value <99 mg/dl) and chemiluminescence kit (reference values 3–16 mcUI/ml), respectively. HOMA- IR index [29], an estimation of insulin resistance, was calculated as fasting insulin (mU/l) x fasting glucose/22.5 (reference value 2.1±0.7). Estradiol was measured by chemiluminescence using an automated XP kit (Siemens Diagnostics; reference values for postmenopausal women without HT were <44 pg/ml). TSH (for exclusion of thyroid dysfunction) was measured by chemiluminescence using an automated Advia Centaur XP kit (Siemens Diagnostics; reference values 0.350–5.900 mcU/ml). High sensitivity C-reactive protein was measured by nefelometry, being considered indicative of low if≤0.099 mg/dl; intermediate if between 0.1 to 0.3 mg/dl and high cardiovascular risk if >0.3 mg/dl. Oxidated LDL was determined by espectofotometry (reference value <0,5 nM/mg ApoPt). Triglycerides, total and HDL cholesterol were determined by enzymatic colorimetric kits (reference values <150 mg/dl, <200 mg/dl and >40 mg/dl, respectively). LDL cholesterol was calculated from previous dosages (reference values <130 mg/dl).

### Nailfold videocapillaroscopy (NVC)

This technique is a non-invasive, accurate and reproducible exam derived from capillaroscopy [30] fully described previously [31]. Briefly, the patient was comfortably seated in a chair with her 4th finger of the left hand positioned at the heart level under the view of a microscope (DM/LM, Leica, Wetzlar, Germany) connected to a CD recorder. Capillaries, microvessels with only the endothelial layer, and red blood cells passing through them were directly visualized and images stored to be further analyzed by another observer, with the help of specific computer assisted-software. Red blood cell velocity (RBCV, mm/s) at rest was used to quantify blood flow and tissue perfusion. A maneuver to elicit NO production was performed, namely 1 min ischemia and subsequent reactive hyperemia response upon occlusion release, eliciting red blood cell velocity to reach a peak (RBCV_max_, mm/s). The time to reach the peak (TRBCV_max_, s) was used to infer the elasticity of the vascular wall. NVC was performed twice in each patient: at baseline (NVC_1_) and, in continuity, 1 hour after acute nasal 17β-estradiol application (NVC_2_). This approved drug for menopause treatment in Brazil acts in a pulsed fashion, with plasma estradiol levels reaching 1,200–1,500 pg/dl up to 30 min after administration, saturating estrogen receptors and returning to postmenopausal values after 2 hours. Succeeding NVC_2_, the patient received breakfast and was discharged.

### DNA extraction and genotyping analysis

Genomic DNA was extracted from peripheral blood leukocytes using a QIAamp DNA Blood Mini Kit following manufacturer’s protocol (Qiagen). All DNA samples were assayed using polymerase chain reaction (PCR) for detection of polymorphisms in *ESR1* (*PvuII* and *XbaI*) and *NOS3 genes* (*VNTR*, *T-786C* and *G894T*). PCR reactions were performed in a final volume of 25 µl, containing approximately 50 ng of genomic DNA, 1 µmol/l of each primer, 0.2 mmol/l dNTPs, and 1.25 U of DNA Polymerase (Life Technologies).

Genotyping of *ESR1* polymorphism was performed using PCR–restriction fragment-length polymorphism (PCR–RFLP) analysis [32]. Genotyping of *PvuII* and *XbaI* polymorphisms was performed using the following oligonucleotide primers: 5′CTG CCA CCC TAT CTGTAT CTT TTC CTATTC TCC3′ (sense) and 5′TCT TTC TCT GCC ACC CTG GCG TCG ATT ATC TGA3′ (antisense). To differentiate *PvuII* and *XbaI* polymorphisms, PCR product of 1,372 bp was digested for 3 h with *PvuII* and *XbaI* restriction enzymes, separately. Digestion products were electrophoresed on 2.5% agarose gel stained with GelRed (Biotium). After *PvuII* digestion, wild-type TT produced fragments of 982, and 390 bp, while mutated homozygous variant CC exhibited one fragment of 1,372 bp. Fragments for *XbaI* digestion were: mutated homozygous variant AA produced fragments of 936, and 436 bp, and the wild-type GG exhibited one fragment of 1,327 bp.

The *VNTR* region of *NOS3* was amplified using the primers 5′AGG CCC TAT GGT AGT GCC TTT3′ (sense) and 5′TCT CTT AGT GCT GTG GTC AC3′ (antisense), as described elsewhere [33]. PCR products were visualized in 4% agarose gels stained with GelRed (Biotium). Alleles obtained when this region was amplified were classified according to number of repeating units. Alleles *c*, *a*, *b*, and *y* were found, consisting of 3, 4, 5, 6 or 7 repeating units. For analysis, only alleles with four or five repeating units were included. For T*-786C* polymorphism of *NOS3*, 282 bp fragment was amplified using primers 5′TGG AGA GTG CTG GTG TAC CCC A3′ (sense) and 5′GCC TCC ACC CCC ACC CTG TC3′ (anti-sense) as described elsewhere [34]. PCR products were then digested for 3 hours by *MspI* and visualized in 4% agarose gels stained with GelRed (Biotium). TT homozygotes had two fragments (140 bp and 40 bp), TC heterozygotes had four DNA bands (194 bp, 149 bp, 88 bp and 45 bp), and CC homozygotes showed three fragments (90 bp, 50 bp, and 40 bp). For *NOS3 G894T* polymorphism, PCR primers were generated to amplify a 248 bp fragment encompassing the missense mutation: 5′AAG GCA GGA GAC AGT GGA TGG A3′ (sense) and 5′CCC AGT CAA TCC CTT TGG TGC TCA3′ (antisense). PCR products were then digested for 16 hours with *BanII* and visualized in 3% agarose gels stained with GelRed (Biotium). As described previously [34], GG homozygotes had two fragments (163 bp and 85 bp), GT heterozygotes had three DNA bands (248 bp, 163 bp and 85 bp), and TT homozygotes showed only one fragment (248 bp).

Hardy-Weinberg equilibrium was determined with use of a χ^2^ goodness-of-fit test on the basis of expected frequencies, calculated using the assumption of Hardy-Weinberg equilibrium. Statistical analysis was performed on each group separately using the Windows software SPSS version 14.0. Failure of data to confirm the normality hypothesis meant that ANOVA or t-tests could not be performed and nonparametric tests, U-Mann Whitney to contrast two samples, and Kruskal-Wallis (with Dunn’s multiple comparison test) to contrast more than two samples were therefore applied. Results were considered significantly different for p<0.05.

## Results

Clinical, videocapillaroscopy and laboratorial data from 100 postmenopausal women, divided into HD (n = 30) and healthy (n = 70) groups, are presented in Table 1. Although many results do not follow a Gaussian distribution, mean and median values were very close. Mean age, time since menopause, intensity of vasomotor symptoms, estradiol levels, previous smoking or oral contraceptive use, and inflammatory markers were similar. As expected, most anthropometric and metabolic analyzed parameters differed between groups. Videocapillaroscopy results were similar between groups, except for time to reach maximal red blood cell velocity before estradiol (TRBCV_max1_), which was shorter in HD women. Estradiol increased at rest and peak RBCV both in HD and healthy women.

**Table 1:** Clinical and laboratory data in HD and healthy women.

	HD			HEALTHY			
Variable	N	Mean ± S.D.	Median (min–max)	N	Mean ± S.D.	Median (min–max)	
Age (years)	30	52.7±4.4	54 (42–59)	70	53.4±7.7	54 (34–70)	0.70
BMI (kg/m^2^)	30	2.9±4.9	26.7 (20–38)	70	25.9±3.7	25.7 (18.8–36.6)	0.070
Time since menopause (years)	30	6.0±4.1	5.5 (1–20)	70	7.0±5.8	6.0 (0.42–24)	0.78
Intensity of vasomotor symptoms (0–10)	30	6.3±3.6	7.0 (0–10)	68	5.8±3.2	6.5 (0–10)	0.36
Oral contraceptives use duration (years)	30	7.3±6.5	7.0 (0–24)	70	6.9±6.5	5.5 (0–21)	0.81
Number of smoked cigarettes in the lifetime	30	203.0±377.6	0 (0–1200)	70	121.4±385.1	0 (0–3000)	0.47
Waist circumference (cm)	30	93.1±11.9	93.5 (70–111)	70	85.9±9.2	85 (69–107)	**0.004**
Systolic blood pressure (SBP, mmHg)	30	144.7±22.1	140 (100–200)	70	121.9±13.8	120 (80–160)	**0.0001**
Diastolic blood pressure (DBP, mmHg)	30	85.7±11.0	85 (60–110)	70	76.1±8.8	80 (50–93)	**0.0001**
RBCV_1_ (mm/s)	29	1.46±0,09	1.46 (1.25–1.68)	70	1.49±0.10	1.49 (1.20–1.74)	0.13
RBCV_max1_ (mm/s)	30	1.66±0.10	1.63 (1.50–1.95)	69	1.69±0.11	1.68 (1.48–2.0)	0.11
RBCV_1_ increment (%)	29	13.5±6.60	12.3 (1.62–31.6)	69	13.2±7.1	11.2 (−1.90–41.7)	0.65
TRBCV_max1_ (s)	30	7.9±1.5	8.0 (4–12)	69	8.7±1.8	9 (4–13)	**0.019**
RBCV_2_ (mm/s)	30	1.69±0.08	1.67 (1.54–1.88)	69	1.69±0.12	1.71 (1.28–2.08)	0.47
RBCV_max2_ (mm/s)	30	1.84±0.08	1.85 (1.69–1.99)	68	1.84±0.12	1.83 (1.63–2.40)	0.78
RBCV_2_ increment (%)	30	9.3±4.4	8.5 (2.8–18.3)	68	9.1±5.7	8.2 (−1.74–29,0)	0.80
TRBCV_max2_ (s)	30	5.9±1.2	6 (4–9)	68	6.1±1.4	6 (2–10)	0.25
Glucose (mg/dl)	30	145.2±66.2	118.5 (82–327)	69	89.4±10.4	90 (69–125)	**0.0001**
HOMA-IR	28	2.59±1,83	2.21 (0.38–7.68)	68	1.31±0.77	1.19 (0.36–3.98)	**0.0002**
Triglycerides, mg/dl	30	168.1±98.2	146.5 (46–406)	68	112.0±51.3	103.5 (40–277)	**0.006**
HDL cholesterol, mg/dl	30	51.8±13.1	51.5 (29–84)	68	57.0±13.8	55.5 (24–95)	0.089
hs C-reactive protein, mg/dl	30	0,42±0,47	0,21 (0,07–1,78)	67	0,29±0,26	0,22 (0,07–1,51)	0,38
Estradiol, pg/ml	30	18.0±11.4	15 (6–56)	67	17.2±11.1	15 (6–75)	0.76
Oxidized LDL, nmol/mg ApoPt	30	0.22±0.08	0.2 (0.1–0.4)	68	0.23±0.08	0.2 (0.1–0.4)	0.99

Reference values: HOMA-IR, 2.1 T 0.7; Triglycerides, <150 mg/dl; HDL cholesterol, >50 mg/dl; hS C-reactive protein, <0.3 mg/dl; Estradiol, <44 pg/ml (post menopause without HT);

Oxidized LDL, <0.5 nmol/mg ApoPt.

BMI, body mass index; HOMA-IR, homeostasis model assessment of insulin resistance; RBCV_1_: red blood cell velocity before estradiol: RBCV_max1_: peak red blood cell velocity before estradiol: RBCV_1_ increment: % of RBCV_max1_ increase in relation to RBCV_1_; TRBCV_max1_: time to reach peak red blood cell velocity before estradiol; RBCV_2_: red blood cell velocity after estradiol: RBCV_max2_: peak red blood cell velocity after estradiol: RBCV_2_ increment: % of RBCV_max2_ increase in relation to RBCV_2_: TRBCV_max2_: time to reach peak red blood cell velocity after estradiol. Comparison between groups was performed by Mann Whitney test.

The frequency of *ESR1* (*PvuII* and *XbaI*) and *NOS3* (*VNTR*, *T-786C* and *G894T*) polymorphisms in HD and healthy women are shown in Table 2. Few variants were not successfully genotyped in every sample, thus numbers reported for specific polymorphism may vary. In most instances, polymorphic homozygotes and heterozygotes were grouped because some variant alleles were underrepresented among groups, hindering statistical analysis. All polymorphisms analyzed did not deviate significantly from Hardy–Weinberg equilibrium in both groups. We found similar distribution of polymorphisms in white and non-white individuals (data not shown), as well as in HD and healthy women, except for *NOS3 VNTR*: the frequency of *aa* genotype was higher among HD women (17.2%) than in the healthy group (3.1%, p = 0.02).

**Table 2:** Frequencies of *ESR1* and *NOS3* polymorphisms in HD and healthy women.

	HD	HEALTHY	
***ESR1***	**n (%)**	**n (%)**	**P value**
***PvuII***			
**TT**	11 (36.7)	24 (34.3)	reference
**TC**	18 (60.0)	36 (51.4)	1.00
**CC**	1 (3.3)	10 (14.3)	0.242
**TC + CC**	19 (63.3)	46 (65.7)	0.823
**Total**	30	70	
***XbaI***			
**AA**	15 (50.0)	37 (52.9)	reference
**AG**	14 (46.7)	30 (42.8)	0.825
**GG**	1 (3.3)	3 (4.3)	1.00
**AG + GG**	15 (50.0)	33 (47.1)	0.830
**Total**	30	70	
***NOS3***	**n (%)**	**n (%)**	**P value**
***VNTR***			
**aa**	5 (17.2)	2 (3.1)	reference
**ab**	8 (27.6)	17 (26.2)	0.091
**bb**	16 (55.2)	46 (70.7)	**0.024**
**ab + bb**	24 (82.8)	63 (96.9)	**0.027**
**Total**	29	65	
***T-786C***			
**TT**	19 (63.3)	33 (47.1)	reference
**TC**	11 (36.7)	32 (45.7)	0.276
**CC**	0	5 (7.2)	0.158
**TC + CC**	11 (36.7)	37 (52.9)	0.190
**Total**	30	70	
***G894T***			
**GG**	19 (63.3)	38 (55.1)	reference
**GT**	11 (36.7)	28 (40.6)	0.658
**TT**	0	3 (4.3)	0.545
**GT + TT**	11 (36.7)	31 (44.9)	0.512
**Total**	30	69	

Comparison between genotypes was performed by Fisher’s exact test.

### *ESR1* polymorphisms associated outcomes

**Figure 1:**
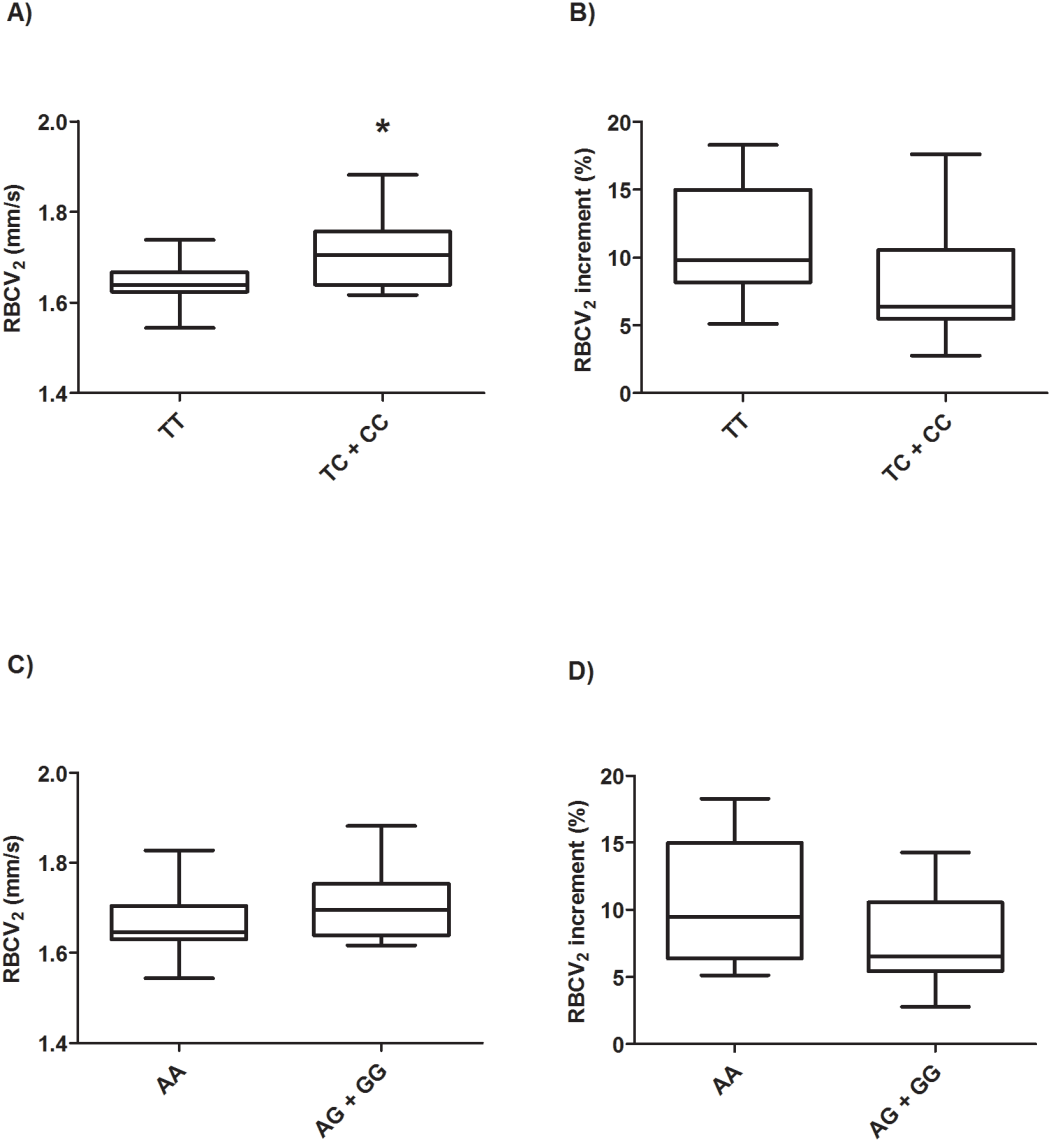
RBCV_2_ and RBCV_2_ increment in HD women according to *ESR1 PvuII* and *Xbal* genotypes. Data are presented as Whiskers plot. Comparison between groups was made by Mann Whitney test. RBCV_2_: Red blood cells velocity (mm/s) after estrogen administration. RBCV_2_ increment: Red blood cells velocity increment (%) after 1 min ischemia and subsequent reactive hyperemia response upon occlusion release after estrogen administration. A and C, RBCV_2_ and *PvuII* and *Xbal* genotypes, respectively. B and D, RBCV_2_ increment and *PvuII* and *Xbal* genotypes, respectively.

When the vasodilatatory response to estradiol was correlated to *ESR1* polymorphisms, we found that HD women who had at least one variant (C) allele of *ESR1 Pvull* polymorphism exhibited higher RBCV_2_ (1.72 *vs* 1.64 mm/s, p = 0.01 compared to TT group, Figure 1A). After estradiol this subgroup of HD women’s RBCV was close to the absolute median value reached by healthy women (1.71 mm/s). In the healthy group, RBCV response after estradiol did not differ in relation to *ESR1 Pvull* polymorphisms.

*ESR1 XbaI* polymorphisms did not influence videocapillaroscopy responses to estradiol in the healthy group (1.69 mm/s for AA and AG/GG groups). RBCV_2_ tended to be higher in HD women harboring at least one G allele, but the results were not statistically significant (1.71 for AG or GG *vs* 1.67 mm/s for AA genotype, p = 0.10, Figure 1C). HD women who had at least one variant allele of the *XbaI* polymorphism (AG or GG genotypes) showed higher HOMA-IR (3.54) than the wild-type homozygous ones (1.64, p = 0.01); healthy women with at least one G allele showed higher glucose levels than the AA subgroup (91.8 vs. 87.1 mg/dl, p = 0.01, Table 3).

**Table 3:** Association of *ESR1* polymorphism (*XbaI*), *NOS3* polymorphism (*G894T*), waist circumference and HOMA-IR in HD and healthy women.

	HD					HEALTHY				
ESR1 polymorphism (XbaI)	AA		AG + GG			AA		AG + GG		
	n	Mean ± SD	n	Mean ± SD	P value	n	Mean ± SD	n	Mean ± SD	P value
Waist circumference (cm)	15	91.1±11.8	15	95.1±12.0	0.42	37	85.8±8.7	33	86.0±9.7	0.85
BMI (kg/m^2^)	15	27.5±5.5	15	28.3±4.5	0.48	37	26.1±3.8	37	25.5±3.7	0.69
Glucose	15	134±72.6	15	156±59.2	0.11	36	87.1±12.1	33	91.8±7.7	**0.01**
HOMA-IR	14	1.64±0.91	14	3.54±2.05	**0.01**	35	1.25±0.69	33	1.36±0.86	0.70
Triglycerides, mg/dL	15	177.6±95.2	15	158.5±103.5	0.38	35	111.7±47.0	33	112.5±56.2	0.72
HDL cholesterol, mg/dL	15	53.5±13.0	15	50.1±13.4	0.52	35	56.9±14.4	33	57.2±13.2	0.99
Estradiol, pg/mL	15	18.3±12.1	15	17.7±11.1	0.97	35	19.2±13.5	32	15.0±7.0	0.30
	**HD**					**HEALTHY**				
**NOS3 polymorphism (G894T)**	**GG**		**GT + TT**			**GG**		**GT + TT**		
	**n**	**Mean ± SD**	**n**	**Mean ± SD**	**P value**	**n**	**Mean ± SD**	**n**	**Mean ± SD**	**P value**
Waist circumference (cm)	19	93.5±13.2	11	92.4±9.7	0.76	38	88.2±8.9	31	83.0±8.9	**0.02**
BMI (kg/m^2^)	19	27.9±5.6	11	27.8±3.7	0.93	38	26.6±4.0	31	24.9±3.2	0.10
Glucose	19	142.1±65.6	11	150.6±70.0	0.67	37	91.0±12.0	31	87.5±8.2	0.28
HOMA-IR	17	2.89±1.56	11	2.13±2.19	0.10	36	1.48±0.87	31	1.09±0.60	**0.05**
Triglycerides, mg/dL	19	178.8±102.4	11	149.5±92.0	0.40	36	116.2±43.6	31	105.3±58.9	0.085
HDL cholesterol, mg/dL	19	50.9±14.0	11	53.2±11.9	0.49	36	57.5±14.8	31	57.0±12.7	0.78
Estradiol, pg/mL	19	15.5±6.2	11	22.4±16.6	0.50	35	19.2±13.3	31	14.9±7.6	0.15

Reference values: HOMA-IR, 2.1 T 0.7; Triglycerides, <150 mg/dl; HDL cholesterol, >50 mg/dl; Estradiol, <44 pg/ml (post menopause without HT).

BMI, body mass index; HOMA-IR, homeostasis model assessment of insulin resistance. Comparison between groups was performed by Mann Whitney test.

### *NOS3* polymorphisms associated outcomes

**Figure 2:**
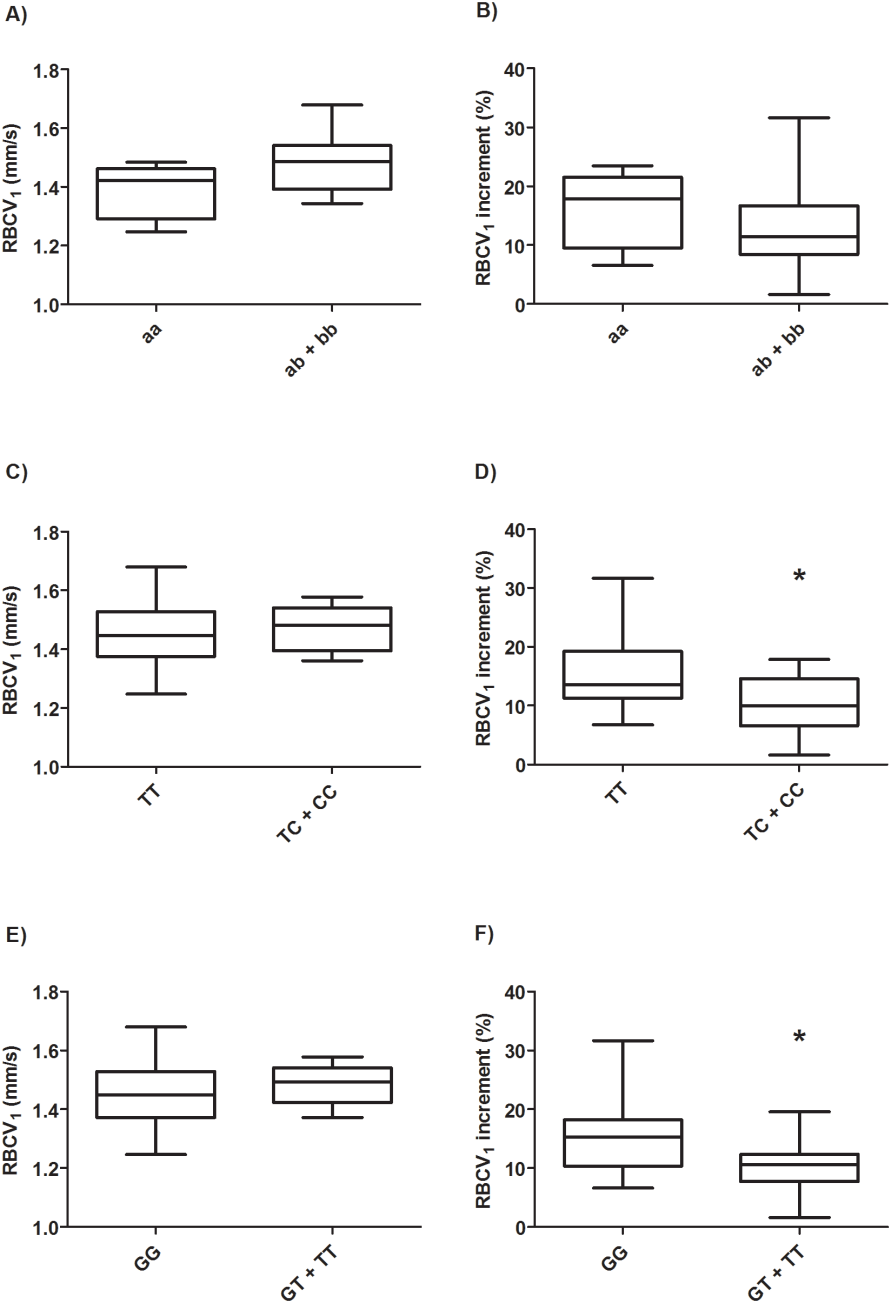
RBCV_1_ and RBCV_1_ increment in HD women according to *NOS3 T-786C and G894T* genotypes. Data are presented as Whiskers plot. Comparison between groups was made by Mann Whitney test. RBCV_1_: Red blood cells velocity (mm/s). RBCV_1_ increment: Red blood cells velocity increment (%) after 1 min ischemia and subsequent reactive hyperemia response upon occlusion release. A, C, and E, RBCV_1_ and *VNTR*, *-786C*, and *G894T* genotypes, respectively. B, D, and F, RBCV_1_ increment and *VNTR*, ***-*** *786C*, and *G894T* genotypes, respectively.

*VNTR NOS3* polymorphisms, which showed different frequencies between HD and healthy women, did not correlate with any studied variables. On the other hand, *NOS3 T-786C* C and *G894T* T alleles were associated with lower RBCV_1_ increments after the reactive hyperemia response in HD women, being these increments of about 15% among wild type and about 10% among those harboring variant alleles (p = 0.02 and 0.04, respectively, Figures 2D and F).

An association with lower HOMA-IR tended to occur in healthy women in relation to T allele in *NOS3 G894T* polymorphism, although this result could be due to a narrower waist circumference in this subgroup (Table 3).

In summary, *ESR1 Pvull, NOS3 T-786C* and *G894T* polymorphisms influenced endothelial-mediated vasodilatatory responses to estradiol and sheer stress, respectively, in early HD postmenopausal women, while these genetic differences seemed not to affect microvascular responses in healthy women.

The presence of G allele in *ESR1 XbaI* was associated with increased insulin resistance in HD patients and higher glucose levels in healthy women. *NOS3 G894T* T allele in healthy women was associated with narrower waist and tendency to lower HOMA-IR.

## Discussion

Endothelial function can be studied through well-validated techniques such as videocapillaroscopy, plethysmograhy and flow mediated dilatation, which assess baseline flow and vasodilatation during the reactive hyperemia response as evidence of NO liberation, upon sheer-stress stimuli obtained after release from induced ischemia. Acute nasal estradiol administration was based on findings that, on cultured human endothelial cells, high transient estradiol levels for one hour were shown to trigger eNOS expression and activity by the same amount observed with 24 h continuous exposure [35]. To know which polymorphisms influence endothelial NO generation following estrogen could help to understand an important endogenous mechanism against initial atherosclerosis progression. Moreover, screening for specific polymorphisms could be useful in predicting how early postmenopausal women with CV risk factors would react in response to estradiol replacement therapy in terms of endothelial function activation and *NO* release as a surrogate marker of future cardiovascular benefits or risks.

Videocapillaroscopy results were similar between HD and healthy women, except for time to reach maximum red blood cell velocity before estradiol (TRBCV_max1_), which was shorter in HD women, as previously described [36], probably due to higher vascular wall stiffness, denoting some degree of atherosclerotic infiltration. Nevertheless, women with CV risk factors in early postmenopausal years showed to retain an endothelial function comparable to healthy ones. Timing of initiation, or window of opportunity, ultimately considered to be between 50–59 years or <10 years after menopause [37], may reflect a critical period of estrogen deficiency not long enough to impair receptor responsiveness by allowing methylation upon disuse. We have to emphasize that all HD women here studied were <60 years old and had a Framingham Score estimating a 10-year risk for coronary heart disease of less than 1%, with low inflammatory markers, supporting that, in initial endothelial dysfunction, microvascular responses to estradiol and sheer-stress may be preserved and enhanced to further halter atherosclerosis injury and progression. In this sense, recent symptomatic postmenopausal women with CV risk factors may benefit from HT vascular and quality of life climacteric symptoms.

HD women who had at least one C allele of *ESR1 Pvull* polymorphism exhibited higher RBCV_2_. *ESR1 Pvull* is one of the most studied polymorphisms described to alter CV risk, yet the mechanisms through which this risk is mediated are not completely understood [38]. Most studies, performed in older male samples, showed that C allele confers more CV risk. When the offspring cohort of the Framingham Heart Study was genetically studied, 1739 unrelated mean age 60 years subjects were followed during 16 years for incident CV events [39]. After adjusting for covariates, the wild, although less common, *ESR1 PvuII CC* genotype was associated with an OR = 2.0 (1.32–3.2, p = 0.004) for major atherosclerotic CVD and an OR = 3.0 (1.7–5.2; p<0.001) for myocardial infarction, while having T allele (*CT* or *TT* genotypes) conferred CV protection. Later on, *ESR1 Pvull* CC genotype was found to be associated with myocardial infarction in different studies in older (>60 years old) men [40], older southern Brazilian subjects [41] and over 1,000 Chinese men [42]. However, estradiol cardiovascular effects may perform differently according to the extent of vascular atherosclerotic disease. Estrogen levels were shown to modulate endothelial ER expression throughout the menstrual cycle [43], so postmenopausal women subjected to HT could present distinct outcomes in relation to their *ESR1* receptor polymorphisms enhancement or down regulation. A Greek team compared the same *ESR1* polymorphisms we have studied in relation to the extent of coronary artery disease (CAD) in 173 older women with various CV risk factors referred to angiography [19]. They found a significant correlation for CC *Pvull* genotype and CAD severity, as well as for the G allele in *XbaI* polymorphismsm, which in our study was linked to insulin resistance but not to NO induced vasodilatation. We have studied early postmenopausal women and a significantly higher vascular response to estradiol was present in around 65% of our HD group, who had at least one C allele of *Pvull*. Our findings provide further evidence that NO bioavailability may be the main mechanism linked to *Pvull* C allele CV protection or hazard, depending on the stage of the atherosclerotic process. A larger NO bioavailability brought by CC or CT *Pvull* genotypes can be translated into interaction of NO with superoxide anions, present in aged microvessels, producing peroxynitrite, a reactive oxygen species (ROS), which causes vasoconstriction instead of vasodilatation [44]. Previous findings that *ESR1 Pvull* CC was associated with increased systolic blood pressure in men and coronary intima thickness in women [38] reinforce NO estrogen-mediated release as an impaired physiological mechanism determined by this genotype.

In HD women *G894T* T and *T-786C* C alleles in *NOS3* polymorphisms were associated with lower RBCV_1_ increments after reactive hyperemia. The first study showing the impact of *NOS3 G894T* T was published in 2007 [45], where human umbilical vein endothelial cells were subjected to shear stress and eNOS protein levels, phosphorylation, and enzyme function were evaluated. Variant T genotypes had lower NO production pre- and post-shear stress due to an altered localization of the variant protein at the caveolae, leading to diminished shear-dependent responses and impaired coordination of eNOS regulatory cycle. Many other studies then aimed to correlate endothelial function and *NOS3* alleles. The Prospective Investigation of the Vasculature in Uppsala Seniors (PIVUS), a population-based cohort study, analyzed 25 SNPs distributed over a region of about 5 kb upstream and 2 kb downstream from the *NOS3* gene [46] while endothelial function was evaluated with brachial artery ultrasound to assess flow-mediated vasodilatation. In this older population *NOS3 G894T* T allele was associated to higher endothelium-dependent vasodilatation in conduit arteries while another study in 230 normotensive male and female Malay individuals between 18 and 40 years [47] found that heterozygotes for the *NOS3 G894T* polymorphism had lower baseline skin perfusion when compared with wild type homozygous (p = 0.029), like in our study. Another Brazilian study [48] is also in accordance with our findings, as in 110 healthy volunteers the polymorphic group presented lower vascular reactivity 120 min after maximal cardiopulmonary exercise. Regarding *NOS3 T-786C* polymorphism, a German proteomic study in human primary cultured endothelial cells compared fluid shear stress (FSS)-induced protein expression. Cells with CC genotype exhibited a greatly reduced FSS-induced eNOS expression as well as diminished NO synthesis capacity when compared to TT cells [49]. Again, results seem to point to caution when interpreting results related to polymorphisms and vascular reactivity, since they are subjected to many variables, like experimental design, biochemical parameters, ethnic differences, but especially age and pre-existing diseases determining baseline degree of vascular damage.

The presence of the G allele in *ESR1 XbaI* was associated to increased insulin resistance in HD patients and higher glucose levels in healthy women. These are important findings, since the use of estrogen alone and estrogen plus progestin in the prospective randomized double blind trial Women’s Health Initiative were associated with decreased risk for type 2 diabetes [37]. To know which woman is at risk for diabetes mellitus type 2, by anthropometrical, laboratorial and now genetic data could help to identify further HT individualized benefits. Estrogen receptors are expressed in non-reproductive tissues, including skeletal and smooth muscle, adipose tissue and pancreas, supporting their involvement with multiple systems [38]. *ESR1* was described to be important for proper glucose homeostasis: female and male αERKO obese mice show adipocyte hyperplasia and insulin resistance, while ERα resistant men present impaired glucose metabolism, insulin resistance, hyperinsulinemia, elevated glycosylated hemoglobin level, and acanthosis nigricans [50]. In male and female mice, estradiol was shown to protect against glucose intolerance induced by high fat diet [51] and also to promote β-pancreatic cell survival by preventing apoptosis through non-classical ER mediated extra-nuclear mechanisms, with predominant ERα effect [52]. Recent papers emphasize non-hepatic actions [53] in adipose tissue or skeletal muscle. Membrane effects are the most implicated mechanisms for estrogen-mediated insulin sensitivity, because estrogen binding to its membrane receptor could influence mitogen-activated protein kinase (MAPK), located on phosphorylated aminoacid residues, signaling the insulin pathway and influencing insulin receptor expression especially on adipocytes [38]. *ESR1 Xbal* G alelle was associated to insulin sensitivity and metabolic syndrome in the Study of Women’s Health Across the Nation [54], but only in Asian ones. Furthermore, *ESR1 Xbal* polymorphism was associated to metabolic syndrome in 548 individuals from 42 African-American families from the Insulin Resistance Atherosclerosis Family Study (OR = 1.53; 1.05–2.27, p = 0.029) [55]. Other studies related *ESR1 Xbal* G allele to lower insulin secretory capacity, suggesting a role on diabetes type 2 installation [38]. However, these findings are not universal, G polymorphisms frequencies may vary across populations, and most importantly, have different consequences depending on age, gender and hormonal status, not allowing for extrapolations. For example, in 500 normoglycemic controls and type 2 diabetic patients *ESR1 XbaI* genotypes were not associated to diabetes or fasting glucose levels in Iranian females, but rather in males (p = 0.02) [56]. Insulin resistance is a complex pathophysiological feature with different organs and targets in the carbohydrate metabolism that can be genetically influenced; in this setting our study reinforces *ESR1 Xbal* polymorphism as a piece in insulin sensitivity.

One of our study strengths was to analyze genetic determinants of estrogen action in early postmenopausal women, since they are the ones who most benefit from HT. Also, we have examined the whole chain involved in endothelial NO generation, namely two *ESR1* and three *NOS3* polymorphisms most implicated in CV disease. Another important point was to include, along with healthy ones, a group of recent postmenopausal women with CV risk factors, in which initial endothelial dysfunction was already installed, and in fact we could see that in their slightly impaired endothelial function the polymorphisms influence was more evident.

The finding that in our HD women, with Framingham Risk Score <1%, the C allele of *PvuII ESR1* was associated to increased endothelial response after estrogen is consistent to the thoughts that later on, in a more damaged endothelium with atherosclerotic infiltration and unstable plaques, this observed beneficial activation could lead to an increase in cardiovascular risk, as seen in older male populations. This phenomenon was described by Vanhoutte in a review article “Endothelium-dependent contractions: when a good guy turns bad!” (57). In brief, Vanhoutte describes the constant balance within the endothelium between the production of relaxing factors (NO being the major one) and of cyclooxygenase-derived vasoconstrictor substances (EDCF, especially prostanoids such as COX). This balance is decisive for blood flow regulation according to physiological necessities like temperature and exercise changes. However, in a model of spontaneous hypertension, a progressively dysfunctional endothelium develops. At an advanced setting, in response to acetylcholine (a known NO synthesis stimulator, through M3 muscarinic endothelial receptors), a concomitant endothelial release of EDCF occurs, impairing or reversing the expected vasodilator response. Endothelium-dependent relaxations in response to acetylcholine are also blunted in diabetes, where high glucose levels result in increased oxidative stress and over- expression of EDCFs such as COX-1 and COX-2. This evidence let us conclude that the mentioned C allele of *Pvull ERS1* is linked to endothelial activation: however, depending on endothelial status, this activation may lead to a predominant vasodilator, neutral or vasoconstrictor effect, a beautiful example of genetic-acquired interaction. These opposite effects may explain why HT cardiovascular outcomes are so conflicting. Women already presenting CV risk factors in early menopause undergoing estrogen therapy must be followed for atherosclerotic progression with care.

The small number of HD patients could be a limitation but in fact we found significant results in this group and even associations between insulin resistance and genetic polymorphisms, which was not our main goal. The small number of individuals was limiting for more sophisticated studies, such as haplotype analysis. Some haplotypes would be so rare that statistical analysis would be impossible. However, the study was designed to have a robust group of clinical and laboratorial data, consisting of women showing similar time since menopause and hormonal profiles. Moreover, this group of women was included in other studies of the group and their vascular responses before and after administration of estradiol were already well established. The Brazilian population is mixed and that could represent a limitation, although we did not find different polymorphism distributions between white and non-white women. Polymorphism frequencies vary between populations and their distribution must be taken into account in each specific community. Compared to previous studies, enhancement of endothelial response to estradiol may represent a benefit or risk, depending on vascular atherosclerotic stage.

## Conclusions

*ESR1 and NOS3* polymorphisms influenced vasodilatatory baseline and after estradiol responses in HD but not in healthy early postmenopausal women. The C variant allele of *ESR1 Pvull* polymorphism was associated to higher RBCV values after estradiol while *G894T and T-786C NOS3* polymorphisms were associated to lower RBCV_1_ reactive hyperemia increment.

In relation to metabolic outcomes, G variant allele of *ESR1 XbaI* polymorphism was associated to higher HOMA-IR in HD women. In healthy women increased waist and HOMA-IR were found in association with G alelle in *NOS3 G894T* polymorphism.

Different estrogen receptors polymorphisms may play a role in CV risk related to different outcomes. When balancing HT risks and benefits in early postmenopausal women with CV risk factors such as diabetes and hypertension, *ESR1 Pvull, NOS3 G894T* and *T-786C* polymorphism analysis may be considered in relation to endothelial-mediated benefits, fundamental in atherosclerosis progression process. Atherosclerotic stage may determine whether endothelial increase NO production after estrogen will produce favorable or unfavorable outcomes. *ESR1 XbaI* and *G894T NOS3* polymorphisms may be useful in accessing insulin resistance and type 2 diabetes risks, even before menopause and occurrence of metabolic disease.

## References

[1] Go AS, Mozaffarian D, Roger VL, Benjamin EJ, Berry JD, American Heart Association Statistics Committee and Stroke Statistics Subcommittee, et al (2013) Executive summary: heart disease and stroke statistics-2013 update: a report from the American Heart Association. Circulation 127: 143–152. 23283859 10.1161/CIR.0b013e318282ab8f

[2] Mikkola TS, Gissler M, Merikukka M, Tuomikoski P, Ylikorkala O (2013) Sex differences in age-related cardiovascular mortality. PLoS One 8: e63347. 23700418 10.1371/journal.pone.0063347 PMC3658978

[3] Nofer JR (2012) Estrogens and atherosclerosis: insights from animal models and cell systems. Journal of Molecular Endocrinology 48: R13–29. 22355098 10.1530/JME-11-0145

[4] Ward BW, Schiller JS (2013) Prevalence of Multiple Chronic Conditions Among US Adults: Estimates From the National Health Interview Survey, 2010. Preventing chronic disease 10: E65. 23618545 10.5888/pcd10.120203 PMC3652717

[5] Moncada S, Higgs A (1993) The L-arginine-nitric oxide pathway. New England Journal of Medicine 329: 2002–2012. 7504210 10.1056/NEJM199312303292706

[6] Channon KM, Guzik TJ (2002) Mechanisms of Superoxide Production in Human Blood Vessels: Relationship to Endothelial Dysfunction, Clinical and Genetic Risk Factors. Journal of Physiology and Pharmacology 53: 515–524. 12512689

[7] Gilchrist M, Shore AC, Benjamin N (2011) Inorganic nitrate and nitrite and control of blood pressure. Cardiovascular Research 15: 492–498. 10.1093/cvr/cvq309 20884639

[8] Masood D, Roach EC, Beauregard KG, Khalil RA (2010) Impact of Sex Hormone Metabolism on the Vascular Effects of Menopausal Hormone Therapy in Cardiovascular Disease. Current Drug Metabolism 11: 693–714. 21189141 10.2174/138920010794233477 PMC3063102

[9] Holm P, Korsgaard N, Shalmi M, Andersen HL, Hougaard P, et al (1997) Significant reduction of the antiatherogenic effect of estrogen by long-term inhibition of nitric oxide synthesis in cholesterol-clamped rabbits. Journal of Clinical Investigation 100: 821–828. 9259581 10.1172/JCI119597 PMC508254

[10] Kim KH, Bender JR (2009) Membrane-initiated actions of estrogen on the endothelium. Molecular and Cellular Endocrinol 308: 3–8. 10.1016/j.mce.2009.03.025 PMC2701909 19549586

[11] Arnal JF, Fontaine C, Billon-Galés A, Favre J, Laurell H, et al (2010) Estrogens receptors and endothelium. Arteriosclerosis, Thrombosis, and Vascular Biology 30: 1506–1512. 10.1161/ATVBAHA.109.191221 20631350

[12] Chow RW, Handelsman DJ, Ng MK (2010) Minireview: rapid actions of sex steroids in the endothelium. Endocrinology 151: 2411–2422. 20392826 10.1210/en.2009-1456

[13] Speroff L (2000) A clinical understanding of the estrogen receptor. Annals of the New York Academy of Sciences 900: 26–39. 10818389 10.1111/j.1749-6632.2000.tb06213.x

[14] Knowlton AA, Lee AR (2012) Estrogen and the cardiovascular system. Pharmacology & Therapeutics 135: 54–70. 22484805 10.1016/j.pharmthera.2012.03.007 PMC5688223

[15] Billon-Galés A, Fontaine C, Douin-Echinard V, Delpy L, Berges H, et al (2009) Endothelial estrogen receptor-α plays a crucial role in the atheroprotective action of 17β-estradiol in low-density lipoprotein receptor-deficient mice. Circulation 120: 2567–2576. 19996016 10.1161/CIRCULATIONAHA.109.898445

[16] Bollig A, Miksicek RJ (2000) An estrogen receptor-α splicing variant mediates both positive and negative effects on gene transcription. Molecular Endocrinology 14: 634–649. 10809228 10.1210/mend.14.5.0460

[17] Lu H, Higashikata T, Inazu A, Nohara A, Yu W, et al (2002) Association of estrogen receptor-alpha gene polymorphisms with coronary artery disease in patients with familial hypercholesterolemia. Arteriosclerosis, Thrombosis, and Vascular Biology 1: 817–823. 10.1161/01.atv.0000014424.18209.21 12006396

[18] Kjaergaard AD, Ellervik C, Tybjaerg-Hansen A, Axelsson CK, Grønholdt ML, et al (2007) Estrogen receptor alpha polymorphism and risk of cardiovascular disease, cancer and hip fracture: Cross sectional, cohort, and case-control studies and a meta-analysis. Circulation 115: 861–871. 17309937 10.1161/CIRCULATIONAHA.106.615567

[19] Alevizaki M, Saltiki K, Cimponeriu A, Kanakakis I, Xita N, et al (2007) Severity of cardiovascular disease in postmenopausal women: Associations with common estrogen receptor alpha polymorphic variants. European Journal of Endocrinology 156: 489–496. 17389465 10.1530/EJE-06-0685

[20] Yaich L, Dupont WD, Cavener DR, Parl FF (1992) Analysis of the PvuII restriction fragment-length polymorphism and exon structure of the estrogen receptor gene in the breast cancer and peripheral blood. Cancer Research 52: 77–83. 1345763

[21] Andersen TI, Heimdal KR, Skrede M, Tveit K, Berg K, et al (1994) Oestrogen receptor (ESR) polymorphisms and breast cancer susceptibility. Human Genetics 94: 665–670. 7989041 10.1007/BF00206961

[22] Marsden PA, Heng HH, Scherer SW, Stewart RJ, Hall AV, et al (1993) Structure and chromosomal localization of the human constitutive endothelial nitric oxide synthase gene. The Journal of Biological Chemistry 268: 17478–17488. 7688726

[23] Li R, Lyn D, Lapu-Bula R, Oduwole A, Igho-Pemu P, et al (2004) Relation of endothelial nitric oxide synthase gene to plasma nitric oxide level, endothelial function, and blood pressure in African Americans. Am J Hypertens 17: 560–567. 15233974 10.1016/j.amjhyper.2004.02.013

[24] Nakayama M, Yasue H, Yoshimura M, Shimasaki Y, Kugiyama K, et al (1999) T-786–>C mutation in the 5′-flanking region of the endothelial nitric oxide synthase gene is associated with coronary spasm. Circulation 99: 2864–2870. 10359729 10.1161/01.cir.99.22.2864

[25] Doshi AA, Ziolo MT, Wang H, Burke E, Lesinski A, et al (2010) Promoter polymorphism of the endothelial nitric oxide synthase gene is associated with reduced mRNA and protein expression in failing human myocardium. Journal of Cardiac Failure 16: 314–319. 20350698 10.1016/j.cardfail.2009.12.013 PMC2848179

[26] Tesauro M, Thompson WC, Rogliani P, Qi L, Chaudhary PP, et al (2000) Intracellular processing of endothelial nitric oxide synthase isoforms associated with differences in severity of cardiopulmonary diseases: cleavage of proteins with aspartate vs. glutamate at position 298. Proceedings of the National Academy of Sciences of the United States of America 97: 2832–2835. 10717002 10.1073/pnas.97.6.2832 PMC16015

[27] Leeson CP, Hingorani AD, Mullen MJ, Jeerooburkhan N, Kattenhorn M, et al (2002) Glu298Asp endothelial nitric oxide synthase gene polymorphism interacts with environmental and dietary factors to influence endothelial function. Circulation Research 90: 1153–1158. 12065317 10.1161/01.res.0000020562.07492.d4

[28] Wilson PWF, D’Agostino RB, Levy D, Belanger AM, Silbershatz H, et al (1998) Prediction of coronary heart disease using risk factor categories. Circulation 97: 1837–1847. 9603539 10.1161/01.cir.97.18.1837

[29] Matthews DR, Hosker JP, Rudenski AS, Naylor BA, Treacher DF, et al (1985) Homeostasis Model Assessment: Insulin Resistance And Beta-Cell Function From Fasting Plasma Glucose And Insulin Concentrations In Man. Diabetologia 28: 412–419. 3899825 10.1007/BF00280883

[30] Ostergren J, Fagrell B (1986) Skin capillary blood cell velocity in man. Characteristics and reproducibility of the reactive hyperemia response. International Journal of Microcirculation Clinical and Experimental 5: 37–51. 3721750

[31] Clapauch R, Mecenas AS, Maranhão PA, Bouskela E (2009) Microcirculatory function in postmenopausal women: role of aging, hormonal exposure and metabolic syndrome. Microvascular Research 78: 405–412. 19695269 10.1016/j.mvr.2009.08.003

[32] Safarinejad MR, Shafiei N, Safarinejad S (2010) Association of polymorphisms in the estrogen receptors alpha, and beta (ESR1, ESR2) with the occurrence of male infertility and semen parameters. The Journal of Steroid Biochemistry and Molecular Biology 122: 193–203. 20599614 10.1016/j.jsbmb.2010.06.011

[33] Tanus-Santos JE, Desai M, Deak LR, Pezzullo JC, Abernethy DR, et al (2002) Effects of endothelial nitric oxide synthase gene polymorphisms on platelet function, nitric oxide release, and interactions with estradiol. Pharmacogenetics 12: 407–413. 12142730 10.1097/00008571-200207000-00008

[34] Safarinejad MR, Safarinejad S, Shafiei N, Safarinejad S (2013) Effects of the T-786C, G894T, and Intron 4 VNTR (4a/b) polymorphisms of the endothelial nitric oxide synthase gene on the risk of prostate cancer. Urologic Oncology 31(7): 1132–1140. 22317880 10.1016/j.urolonc.2012.01.002

[35] Simoncini T, Fornari L, Mannella P, Varone G, Caruso A, et al (2005) Differential estrogen signaling in endothelial cells upon pulsed or continuous administration. Maturitas 50: 247–258. 15780523 10.1016/j.maturitas.2004.04.001

[36] Clapauch R, Mecenas AS, Maranhão PA & Bouskela E (2012) Early postmenopausal women with cardiovascular risk factors improve microvascular dysfunction after acute estradiol administration. Menopause 19: 672–679. 22314638 10.1097/gme.0b013e31823a8f43

[37] Santen RJ, Allred DC, Ardoin SP, Archer DF, Boyd N, Endocrine Society, et al (2010) Postmenopausal hormone therapy: an Endocrine Society scientific statement. The Journal of Clinical Endocrinology and Metabolism 95: s1–s66. 20566620 10.1210/jc.2009-2509 PMC6287288

[38] Casazza K, Page GP, Fernandez JR (2010) The association between the rs2234693 and rs9340799 estrogen receptor alpha gene polymorphisms and risk factors for cardiovascular disease: a review. Biological Research for Nursing 12: 84–97. 20702456 10.1177/1099800410371118

[39] Shearman AM, Cupples L, Demissie S, Peter I, Schmid CH, et al (2003) Association Between Estrogen Receptor α Gene Variation and Cardiovascular Disease. JAMA 290: 2263–2270. 14600184 10.1001/jama.290.17.2263

[40] Shearman AM, Cooper JA, Kotwinski PJ, Miller GJ, Humphries SE, et al (2006) Estrogen receptor alpha gene variation is associated with risk of myocardial infarction in more than seven thousand men from five cohorts. Circulation Research 98: 590–592. 16484614 10.1161/01.RES.0000210578.62102.a6

[41] Almeida S, Hutz MH (2006) Estrogen receptor 1 gene polymorphisms and coronary artery disease in the Brazilian population. Brazilian Journal of Medical and Biological Research 39: 447–454. 16612467 10.1590/s0100-879x2006000400004

[42] Shen C, Chen J, Fan S, Li Z, Hu Y, et al (2012) Association between the polymorphism of estrogen receptor α and coronary artery disease in a Chinese population. European Journal of Internal Medicine 23: 175–178. 22284250 10.1016/j.ejim.2011.05.006

[43] Gavin KM, Seals DR, Silver AE, Moreau KL (2009) Vascular Endothelial Estrogen Receptor Is Modulated by Estrogen Status and Related to Endothelial Function and Endothelial Nitric Oxide Synthase in Healthy Women. Journal of Clinical Endocrinology and Metabolism 94: 3513–3520. 19509105 10.1210/jc.2009-0278 PMC2741709

[44] Rodríguez-Mañas L, El-Assar M, Vallejo S, López-Dóriga P, Solís J, et al (2009) Endothelial dysfunction in aged humans is related with oxidative stress and vascular inflammation. Aging Cell 8(3): 226–238. 19245678 10.1111/j.1474-9726.2009.00466.x

[45] Joshi MS, Mineo C, Shaul PW, Bauer JA (2007) Biochemical consequences of the NOS3 Glu298Asp variation in human endothelium: altered caveolar localization and impaired response to shear. The FASEB Journal 21: 2655–2663. 17449720 10.1096/fj.06-7088com PMC7460804

[46] Ingelsson E, Syvanen AC, Lind L (2008) Endothelium-dependent vasodilation in conduit and resistance vessels in relation to the endothelial nitric oxide synthase gene. Journal of Human Hypertension 22: 569–578. 18463668 10.1038/jhh.2008.37

[47] Rasool AIG, Ghazali DM, Abdullah H, Halim AS, Wong AR (2009) Endothelial nitric oxide synthase G894T gene polymorphism and response to skin reactive hyperemia Microvascular Research. 78: 230–234. 10.1016/j.mvr.2009.05.005 19481100

[48] Neves FJ, Silva BM, Rocha NG, Salesa ARK, Ribeiro GS, et al (2010) Effect of the 894G>T polymorphism of the endothelial nitric oxide synthase on vascular reactivity following maximal dynamic exercise Journal of Hypertension. 28: 764–770. 10.1097/HJH.0b013e328334f55c 19952778

[49] Asif AR, Oellerich M, Armstrong VW, Hecker M, Cattaruzza M (2009) T-786C Polymorphism of the nos-3 Gene and the Endothelial Cell Response to Fluid Shear Stress - A Proteome Analysis. Journal of Proteome Research 8: 3161–3168. 19320461 10.1021/pr800998k

[50] Nilsson S, Gustafsson JA (2011) Estrogen Receptors: Therapies Targeted to Receptor Subtypes. Clinical Pharmacology and Therapeutics 89: 44–55. 21124311 10.1038/clpt.2010.226

[51] Riant E, Waget A, Cogo H, Arnal JF, Burcelin R, et al (2009) Estrogens protect against high-fat diet-induced insulin resistance and glucose intolerance in mice. Endocrinology 150: 2109–2117. 19164473 10.1210/en.2008-0971

[52] Liu S, Mauvais-Jarvis F (2009) Rapid, nongenomic estrogen actions protect pancreatic islet survival. Islets 1: 273–275. 20634925 10.4161/isl.1.3.9781 PMC2903892

[53] Matic M, Bryzgalova G, Gao H, Antonson P, Humire P, et al (2013) Estrogen signalling and the metabolic syndrome: targeting the hepatic estrogen receptor alpha action. PLoS One 8: e57458. 23451233 10.1371/journal.pone.0057458 PMC3581463

[54] Lo JC, Zhao X, Scuteri A, Brockwell S, Sowers MR (2006) The association of genetic polymorphisms in sex hormone biosynthesis and action with insulin sensitivity and diabetes mellitus in women at midlife. American Journal of Medicine 119: S69–S78. 10.1016/j.amjmed.2006.07.009 16949391

[55] Gallagher CJ, Langefeld CD, Gordon CJ, Campbell JK, Mychaleckyj JC, et al (2007) Association of the estrogen receptor-alpha gene with the metabolic syndrome and its component traits in African-American families: the Insulin Resistance Atherosclerosis Family Study. Diabetes 56: 2135–2141. 17513703 10.2337/db06-1017

[56] Meshkani R, Saberi H, MohammadTaghvaei N, Tabatabaiefar MA (2012) Estrogen receptor alpha gene polymorphisms are associated with type 2 diabetes and fasting glucose in male subjects. Molecular and Cellular Biochemistry 359: 225–233. 21837403 10.1007/s11010-011-1017-9

[57] Vanhoutte PM, Tang EH (2008) Endothelium-dependent contractions: when a good guy turns bad! J Physiol. 586(Pt22): 5295–5304. 10.1113/jphysiol.2008.161430 PMC2655387 18818246

